# An Ultra-Sensitive Colorimetric Sensing Platform for Simultaneous Detection of Moxifloxacin/Ciprofloxacin and Cr(III) Ions Based on Ammonium Thioglycolate Functionalized Gold Nanoparticles

**DOI:** 10.3390/s25103228

**Published:** 2025-05-21

**Authors:** Lihua Zhang, Jiang Li, Juan Wang, Xu Yan, Jinping Song, Feng Feng

**Affiliations:** 1 Shanxi Provincial Key Laboratory of Chemical Biosensing, School of Chemistry and Chemical Engineering, Shanxi Datong University, Datong 037009, China; lihuazhang888@163.com (L.Z.); possumlee@126.com (J.L.); juanwang0422@163.com (J.W.); xuyan04032025@163.com (X.Y.); 2 Department of Chemistry, Xinzhou Normal University, Xinzhou 034000, China; songjphx@163.com

**Keywords:** colorimetric chemosensor, ammonium thioglycolate, moxifloxacin (MOX), ciprofloxacin (CIP), Cr(III), gold nanoparticles

## Abstract

**Highlights:**

**What is the main findings?**

- ATG-functionalized AuNPs were prepared as a colorimetric chemosensor with ultra-sensitivity for the simultaneous detection of MOX/CIP and Cr(III).

**What are the implications of the main findings?**

- ATG-AuNPs sensor color transitions from wine-red to gray-blue indicated successful detection.
- The detection limits for MOX/CIP and Cr(III) are 1.57 µM, 1.30 µM, and 57.1 nM, respectively.
- The presented method was used to detect multiple analytes in lake and tap water samples.

**Abstract:**

Water pollution by antibiotics and heavy metals threatens the ecological environment and human health globally, yet there is no rapid method to detect multiple antibiotics and metal ions simultaneously. A simple, fast, and ultra-sensitive colorimetric chemosensor for the simultaneous detection of moxifloxacin (MOX), ciprofloxacin (CIP), and Cr(III) based on the aggregation of ammonium thioglycolate (ATG)-functionalized gold nanoparticles (ATG-AuNPs) was developed. Following the addition of MOX, CIP, and Cr(III), a color change in the solution was observed from wine-red to blue-grey. The UV–Vis signal of the ATG-AuNPs system blended with MOX, CIP, and Cr(III) in the range of 0~200 µM, 0~100 µM, and 0~5 µM was assessed and measured with detection limits (LODs) of 1.57 µM, 1.30 µM, and 57.1 nM calculated by 3σ/S, respectively. Therefore, this system has the potential to act as an effective colorimetric chemosensor for simultaneously detecting MOX, CIP, and Cr(III) in complex environmental systems.

## 1. Introduction

Antibiotics and heavy metal pollution pose significant risks to public health and the environment worldwide. Moxifloxacin (MOX) and ciprofloxacin (CIP) are popular broad-spectrum quinolone antibiotics exhibiting high activity against various bacterial species, both Gram-negative and Gram-positive [1,2]. They have been employed in treating severe-to-moderate community intravenous pneumonia, infections of the skin and soft tissues, acute sinusitis with bacteria caused by susceptible microorganisms, and acute bacterial chronic bronchitis [3]. They have also been used for diseases caused by bacteria resistant to other antibiotics, such as aminoglycosides and β-lactams [4]. As a result, large amounts of residues of MOX and CIP are inevitably discharged into the surrounding environment and food, subsequently accumulating in the human body via the biological cycle; uncontrolled utilization of MOX and CIP may induce human diseases and result in their excretion in an unchanged form through urine, leading to considerable detrimental effects on human health and environmental issues [5,6]. Organizations and countries have established maximum residue limits (MRLs) to reduce the misuse of antibiotics in various food products. For instance, India has set a maximum residue limit of 1.0 µg/kg for MOX in food samples. Moreover, the European Union has established a maximum residue limit (MRL) for certain fluoroquinolones, including CIP, in edible animal products, set at 30 µg·kg^−1^ for various edible animal tissues [7]. In addition, the trivalent chromium ion (Cr(III)) is another molecule that poses a health risk to humans, being one of the most prevalent heavy metal ions in industrial effluent, and has the potential for accumulation in the human body through the food chain [8,9]. Although Cr(III) is a vital micronutrient that can efficiently sustain the metabolic functions of carbohydrates, adipose tissues, and proteins in the human body [10], an excessive intake of Cr(III) can bind robustly to DNA in human cells, negatively changing their cellular structure and composition and thereby elevating the risks of DNA damage and cancer [11]. Considering the complexity of sample detection environments, as mentioned above, there is an urgent need to develop a new simultaneous detection method that possesses the advantages of being simple, cheap, and having high sensitivity for simultaneous identification and distinguishing between MOXI/CIP and Cr(III) by a standalone system.

Up until now, a variety of plentiful approaches have been developed to detect MOX/CIP or metal ions, such as high-performance liquid chromatography (HPLC) [12,13,14], liquid chromatography–mass spectroscopy (LC-MS) [15,16,17], gas chromatography–mass spectroscopy (GC-MS) [18], inductively coupled plasma–mass spectrometry (ICP-MS) [19,20], electrochemical methods [21,22,23], gas atomic absorption spectrometry (AAS) [24,25], and fluorescence detection [26,27,28]. However, these approaches are costly, time-consuming, and laborious and demand sophisticated analysis procedures, restricting their use in the field. Consequently, developing a simultaneous detection system that is straightforward, rapid, inexpensive, and yet offers enhanced performance is essential.

Among the detection techniques, colorimetric sensors have attracted considerable attention since they facilitate simple, rapid, and real-time measurement without sophisticated apparatus [29]. Gold nanoparticles (AuNPs) are frequently employed as colorimetric reporters due to their distance-dependent optical properties [30]. The extinction spectra of AuNPs and their absorbed and dispersed light wavelength depend on the interparticle distance [31]. Therefore, systems based on the analyte-driven aggregation of AuNPs have been utilized for the colorimetric sensing of specific analytes through detection reporters on the AuNPs [32,33,34,35,36,37,38,39]. Notably, the ability of a single sensor to simultaneously detect multiple analytes has recently gained significant attention, owing to advantages including cost reduction and improved analytical efficiency [40,41,42,43,44]. Consequently, the design and synthesis of colorimetric sensors exhibiting high selectivity and sensitivity toward MOX, CIP, and Cr(III) have attracted increasing attention [45,46,47]. Recent advances have led to the development of several colorimetric chemical sensors capable of detecting MOX, CIP, and Cr(III) in diverse industrial applications [48,49,50,51]. Nevertheless, conventional detection methods for MOX, CIP, and Cr(III) are typically performed separately. To date, the simultaneous detection of these three analytes using a single molecular platform has rarely been documented to the best of our knowledge. To address this limitation, we designed and synthesized a novel ammonium thioglycolate (ATG)-functionalized AuNP-based system and systematically evaluated its multi-analyte recognition capabilities. This system is anticipated to function as a versatile multi-analyte recognition sensor in metal ion detection, providing the simultaneous identification of multiple analytes within a single assay.

Herein, we proposed a novel platform comprising ammonium thioglycolate-functionalized AuNPs (ATG-AuNPs) that were employed as a colorimetric chemosensor based upon the aggregation of ATG-AuNPs upon binding to MOX, CIP, and Cr(III). This resulted in the color of the AuNPs changing from wine-red to blue-gray. The influencing factors were optimized to achieve the maximum sensitivity of the prober. Then, its analytical performances were assessed and confirmed before being used to detect MOX/CIP and Cr(III) in actual samples. Finally, the exploited sensing strategy will simultaneously sense multiple targets in realistic samples, paving the way for the potential application of AuNPs or other metal nanoparticles in the identification and differentiation of several targets, which has yet to be published.

## 2. Materials and Methods

### 2.1. Materials

Hydrogen tetrachloroaurate hydrate (HAuCl_4_·4H_2_O), ciprofloxacin (C_17_H_18_FN_3_O_3_), trisodium citrate dihydrate (C_6_H_5_Na_3_O_7_·3H_2_O), and ammonium thioglycolate (C_2_H_7_NO_2_S) were purchased from Aladdin Reagent Co., Ltd. (Shanghai, China). Moxifloxacin (MOX) hydrochloride (C_21_H_24_FN_3_O_4_), tetracycline (TC), and other antibiotics were supplied by Shanghai Macklin Biochemical Co., Ltd. (Shanghai, China). Chromium chloride hexahydrate (CrCl_3_·6H_2_O) and other metal ions were purchased from Tianjin Guangfu Fine Chemical Institute (Tianjin, China). All aqueous solutions were prepared using ultra-pure water (18.2 MΩ, Miui-Q, Millipore) (Dubuque, IA, USA). All the reagents were of analytical grade unless otherwise indicated.

### 2.2. Apparatus

Transmission electron microscope (TEM) images were characterized using a JEM-1011 (Tokyo, Japan) operating at an accelerated voltage of 120 kV. Subsequently, the UV–visible absorbance spectra (UV–Vis) were measured on a Perkin Elmer Lambda 35 Spectrometer) (Waltham, MA, USA) at room temperature in the 200~800 nm wavelength range.

### 2.3. Synthesis of AuNPs

According to the method reported by Frens [52], AuNPs with a 13 nm diameter were prepared by reducing HAuCl_4_ with citrate in an aqueous solution. All glassware utilized in the subsequent experimental process was precleaned in a bath of freshly-generated HNO_3_-HCl (1:3, *v*/*v*), washed successively with distilled water, and air-dried. Briefly, 1.0 mM AuNPs (100 mL) was injected into a 250 mL flask with a magnetic stirrer and heated to boiling. After that, 10 mL sodium citrate (38.8 mM) was quickly added into the above flask. Heating was stopped when a wine-red color was acquired, and then it was stirred at 125 °C for 30 min and allowed to cool to room temperature with successive stirring.

### 2.4. Modification of AuNPs by ATG

To modify the surface of the AuNPs by ATG, 160 µL AuNPs solution was added into a mixed solvent of 4 µL of ATG (4 mM) and 26 μL double-deionized water, and the self-assembly reaction was performed at 400 rpm for 1 h at room temperature to produce ATG-AuNPs.

### 2.5. Colorimetric Detection

In brief, the ATG-AuNPs solution (164 µL) and 10 µL of different MOX, CIP, and Cr(III) concentrations were blended and reacted for 1 min at room temperature with shaking. Then, the absorbance ratio of A_600/_A_520_, A_640/_A_520_, and A_650/_A_520_ was recorded by UV–Vis absorbance spectra. Furthermore, the specificity of the ATG-AuNPs was evaluated by testing with other antibiotics and ions, including Tetracycline (TC), Pefloxacin (PEF), Lomefloxacin Hydrochloride (LXN), Ampicillin trihydrate (AMP), Amoxicillin trihydrate (AMX), Oxytetracycline (OTC), Doxycycline hyclate (DOX), Gibberellic acid (GA), Azithromycin (AZM), Roxithromycin (ROX), Chloramphenicol (CM), Cr(III), Al(III), Mn(II), Pb(II), Cu(II), Hg(II), Ni(II), Fe(II), Ag(I), Fe(III), Zn(II), Ca(II), Ba(II), and Mg(II) (the concentration of all antibiotics and metals ions is 200 μM and 10 μM, respectively). The visual color changes were observed by the naked eye, and images of the samples were captured using a smartphone and subsequently recorded by UV–Vis spectra.

### 2.6. Detection of Real Samples

The applicability of ATG-AuNPs in actual environmental samples was evaluated. The tap water was taken from our laboratory, while the lake water was obtained from the Wenying Lake in Datong City, Shanxi Province. The real samples were pre-treated by centrifuging at 12,000 rpm for 10 min, and then filtered with a 0.22 µm filter to eliminate insoluble matter. The ATG-AuNPs and the real water samples with three analytes were mixed and reacted for 1 min, and the color changes were recorded by UV–Vis absorbance spectra.

## 3. Results and Discussion

### 3.1. Preparation, Characterization, and Detection Mechanism of ATG-AuNPs

**Scheme 1:**
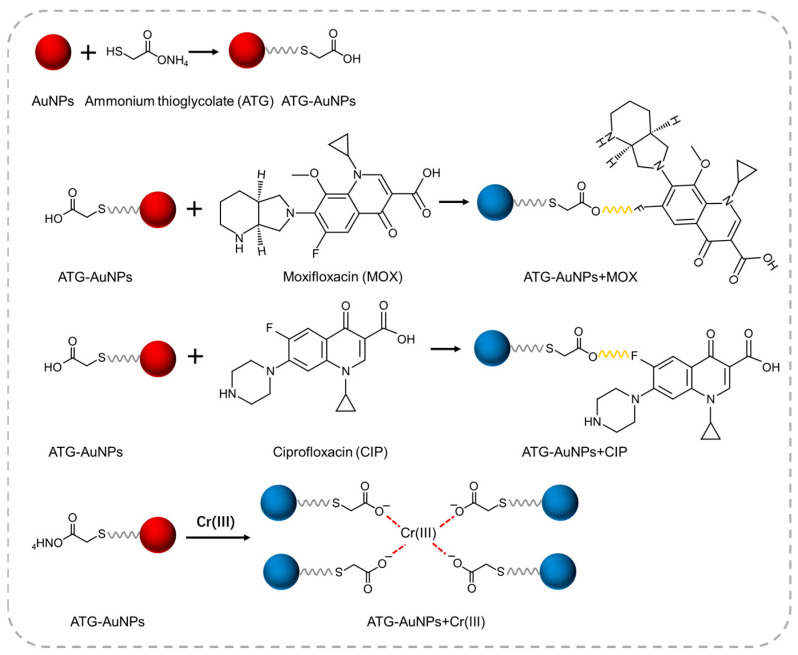
Schematic illustration of a possible mechanism for the colorimetric chemosensing of MOX/CIP and Cr(III).

**Figure 1:**
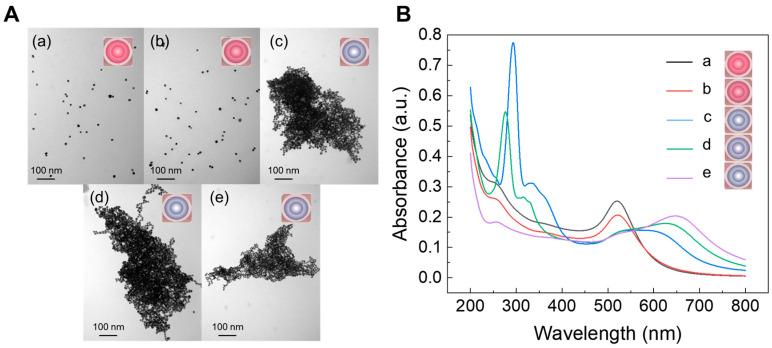
(**A**) TEM image and (**B**) UV–Vis absorption spectra of (**a**) AuNPs, (**b**) ATG-AuNPs, (**c**) ATG-AuNPs+MOX, (**d**) ATG-AuNPs+CIP, and (**e**) ATG-AuNPs+Cr(III).

The possible detection mechanism of MOX/CIP and Cr(III) using ATG-AuNPs is shown in Scheme 1. The AuNPs employed in this study were synthesized via the well-established citrate-reducing approach, and then the AuNPs were modified and stabilized by the ATG ligands. This is likely because the Au-S bond was utilized to bind ATG on the surface of the AuNPs via a self-assembly reaction, similar to that previously reported in the paper [53]. After adding MOX and CIP containing fluorine in their chemical structure, the fluorine can interact with the carboxyl group of ATG selectively, induce aggregation of the ATG-AuNPs, and change the color from wine-red to blue-gray. In addition, in the presence of Cr(III), the carboxyl group of ATG can effectively capture and chelate Cr(III) ions, forming stable octahedral hexacoordinate complexes (excluding two solvent water molecules) [54,55,56]. This coordination interaction induces the aggregation of the ATG-AuNPs, along with a color change. As shown by transmission electron microscopy (TEM), the AuNPs and ATG-AuNPs displayed a nearly spherical shape, uniform particle size, and excellent dispersion. The average diameter of AuNPs is approximately 13 nm (Figure 1A(a,b)). Owing to their capacity to undergo interparticle surface plasmon coupling, the obtained diameter was appropriate for the colorimetric detection (>3.5 nm), resulting in a noticeable color change from wine-red to blue-gray at the level of the nanomolar concentration. Once MOX/CIP and Cr(III) were added to a dispersion of the ATG-AuNPs nanoprobes, the rigid structure formed was insufficient to stabilize the AuNPs solution, leading to the aggregation of AuNPs (Figure 1A(c–e)).

The UV–Vis absorption spectrum was further employed to evaluate the surface plasmon resonance of the AuNPs before and after modification and the optical properties of the modified AuNPs. As illustrated in Figure 1B(a), AuNPs have a sharp absorption peak of 518 nm, a characteristic plasmon resonance absorption band. As expected, the absorption spectra of ATG-AuNPs were similar to those of AuNPs, and the color of the as-synthesized ATG-AuNPs solution still remained wine-red, suggesting that AuNPs do not aggregate after modification (Figure 1B(b)). Significantly, the absorption peaks of ATG-AuNPs were red-shifted from 518 nm to 520 nm. These results concur with previous studies [57,58] indicating that the peak location of surface plasmon absorption is moderately red-shifted owing to changes in the refractive index around the AuNPs by surface functionalization. Upon adding MOX and CIP, the characteristic peak of the ATG-AuNPs at 520 nm diminished markedly, and a new peak at around 600 and 640 nm increased significantly, as illustrated in Figure 1B(c,d). Moreover, the presence of Cr(III) caused a notable decline in the characteristic peak of ATG-AuNPs at 520 nm, accompanied by an increase in a new peak at approximately 650 nm, as shown in Figure 1B(e). Meanwhile, the color changed from wine-red to blue-gray. The above results suggested that the modifier was successfully coated on the surface of the AuNPs to prepare the colorimetric sensors. In addition, the ATG-AuNPs have stable optical features (Figure S1), which is advantageous for analyzing MOX/CIP and Cr(III).

### 3.2. Optimization of the Detection System

The parameters, including the modifier concentration and reaction time, were optimized to identify the optimal conditions for achieving enhanced responses from ATG-AuNPs as a colorimetric probe for identifying and determining MOX/CIP and Cr(III).

#### 3.2.1. The Effect of Concentration of ATG as a Modifier

**Figure 2:**
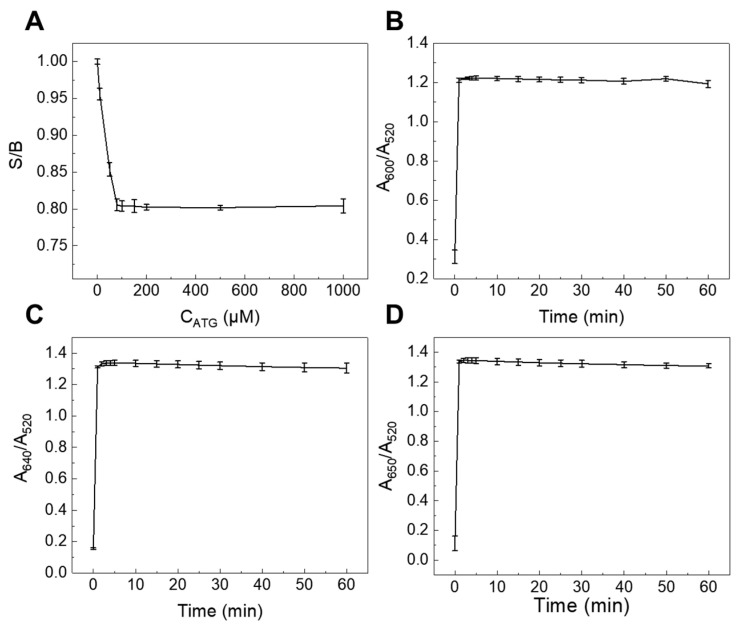
(**A**) The effect of concentrations of ATG (0~1000 µM) on the responsiveness of the AuNPs. The effect of reaction time on the responsiveness of the ATG-AuNPs sensor for MOX(200 µM) (**B**), CIP(100 µM) (**C**), and Cr(III) (10 µM) (**D**).

As mentioned above, the concentration of the modifier is a crucial factor in colorimetric sensing devices, and it should be optimized. The optimal concentration of ATG should consider that not only is sufficient ATG needed to saturate the surface of AuNPs, but no free ATG is distributed in the solution. In this study, 160 µL of AuNPs was mixed with various amounts of ATG to determine the concentration of ATG. As shown in Figure 2A, the absorption of AuNPs at 520 nm decreased significantly with increasing ATG concentrations up to 80 µM, which could be ascribed to the existence of free ATG molecules. As anticipated, with the increasing ATG concentration, the absorption of ATG-AuNPs at 520 nm displayed a negligible change, indicating that almost the entire surface of the AuNPs was adsorbed completely by ATG molecules. This is likely because the -SH groups on the surface of the AuNPs have become saturated. Based on the above results, the optimal concentration value for ATG was 80 µM and this was used for subsequent experiments.

#### 3.2.2. The Effect of the Reaction Time

Response time is another critical metric for evaluating colorimetric chemosensors. The response time of ATG-AuNPs to MOX was determined by observing the change in color with reaction time. A color change was immediately observed when MOX was added to the ATG-AuNPs solution. The absorbance ratio versus the concentration of MOX (200 µM) showed an increasing sensitivity with the incubation time from 0 to 1 min (Figure 2B). However, it remained unchanged for longer periods (Figure S3). That means 1 min was adequate for the reaction of MOX and ATG. Additionally, the UV–Vis absorbance spectra of the reaction of CIP and Cr(III) with the ATG-AuNPs sensor were recorded for 60 min (Figures S4 and S5). The results revealed that the aggregation of ATG-AuNPs in the presence of CIP and Cr(III) was elevated up to 1 min and maintained unchanged (Figure 2C,D). Therefore, all the following tests for CIP and Cr(III) were performed with a responsive time of 1 min, respectively.

Furthermore, the aggregation of the ATG-AuNPs reduced the absorbance at a wavelength of 520 nm while increasing it at 600~650 nm. The sensitivity dependent on MOX reached a maximal value at A_600/_A_520_ and remained unchanged at the larger wavelength (Figure S3). Therefore, the detection wavelengths were determined to be A_600/_A_520_. Additionally, the detection wavelengths for CIP and Cr(III) were fixed at A_640/_A_520_ and A_650/_A_520_, respectively (Figures S4 and S5).

#### 3.2.3. Selectivity Evaluation

**Figure 3:**
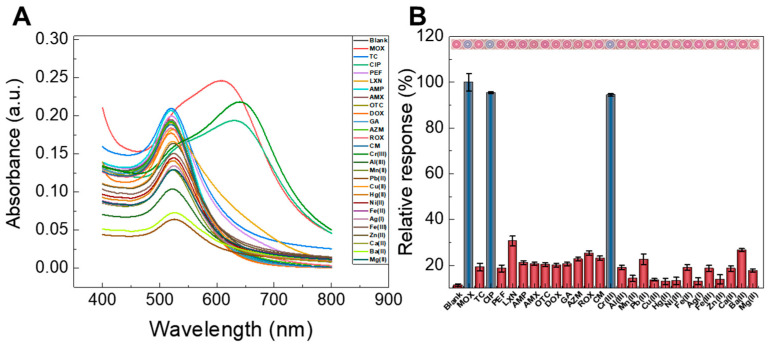
Selectiveness of the detection system. (**A**) UV–Vis absorption spectra and (**B**) the relative response of ATG-AuNPs towards MOX tested against some potential targets; inset: photos showing the corresponding sample color. MOX, TC, CIP, PEF, LXN, AMP, AMX, OTC, DOX, GA, AZM, ROX, and CM are 200 μM, respectively, and Cr(III), Al(III), Mn(II), Pb(II), Cu(II), Hg(II), Ni(II), Fe(II), Ag(I), Fe(III), Zn(II), Ca(II), Ba(II), and Mg(II) are 10 μM, respectively. (Blue-gray color: aggregation; wine-red color: dispersion).

To detect all types of water samples with complicated environments, a high-selectivity measurement assay is desired. To this end, 164 µL ATG-AuNPs was mixed with 10 µL MOX (200 µM) or different other antibiotics (200 µM) and metal ion (10 µM) solutions and incubated for 1 min. The investigated antibiotics and metal ions solutions include TC, CIP, PEF, LXN, AMP, AMX, OTC, DOX, GA, AZM, ROX, CM, Cr(III), Al(III), Mn(II), Pb(II), Cu(II), Hg(II), Ni(II), Fe(II), Ag(I), Fe(III), Zn(II), Ca(II), Ba(II), and Mg(II). As shown in Figure 3A,B, the addition of MOX, CIP, and Cr(III) to the ATG-AuNPs showed an apparent decrease at 520 nm and a new red-shifted peak at 600, 640, and 650 nm by UV–Vis absorbance spectra. Moreover, the presence of MOX/CIP and Cr(III) led to a color change of the ATG-AuNPs from wine-red to blue-gray. In contrast, other antibiotics and metal ions do not result in significant red-shift and color variations relative to the blank of ATG-AuNPs, which is ascribed to the high selectivity of ATG-AuNPs for MOX/CIP and Cr(III) due to the combining action of the carboxyl group of ATG on AuNPs.

#### 3.2.4. Interference Study with Other Substances

**Figure 4:**
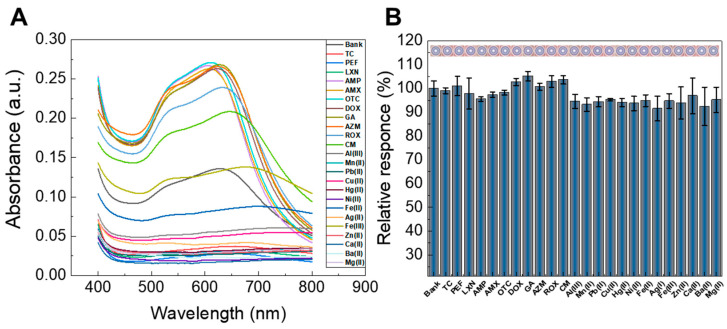
Anti-interference test for the detection system. (**A**) UV–Vis absorption spectra and (**B**) the relative response for the ATG-AuNPs system in the presence of MOX/CIP and Cr(III) and a mixture of MOX/CIP, Cr(III), and other potential targets; inset: photos showing the corresponding sample color. MOX, CIP, TC, PEF, LXN, AMP, AMX, OTC, DOX, GA, AZM, ROX, and CM are 200 μM, respectively, and Cr(III), Al(III), Mn(II), Pb(II), Cu(II), Hg(II), Ni(II), Fe(II), Ag(I), Fe(III), Zn(II), Ca(II), Ba(II), and Mg(II) are 10 μM, respectively.

To investigate the anti-interference ability of other substances on the performance of the ATG-AuNPs system for MOX/CIP and Cr(III), the determination and discrimination of three or two analytes were investigated in the presence of TC, PEF, LXN, AMP, AMX, OTC, DOX, GA, AZM, ROX, CM, Al(III), Mn(II), Pb(II), Cu(II), Hg(II), Ni(II), Fe(II), Ag(I), Fe(III), Zn(II), Ca(II), Ba(II), and Mg(II). Figure 4 illustrates the percentage response together with the color change of the ATG-AuNPs sensor for various compounds in the addition of MOX (200 µM)/CIP (200 µM) and Cr(III) (10 µM), MOX/Cr(III) (Figure S6), as well as MOX/CIP (Figure S7), as the aggregation of the nanoparticles is evident from the observed results and their color change occurred with the addition of different types target analytes, suggesting that the formation of chemical bonds between these analytes and ATG is much more robust than the other samples above. Therefore, the developed sensor is a valuable and reliable method for quantitatively measuring MOX/CIP and Cr(III) in actual samples.

#### 3.2.5. Sensitive Detection

**Figure 5:**
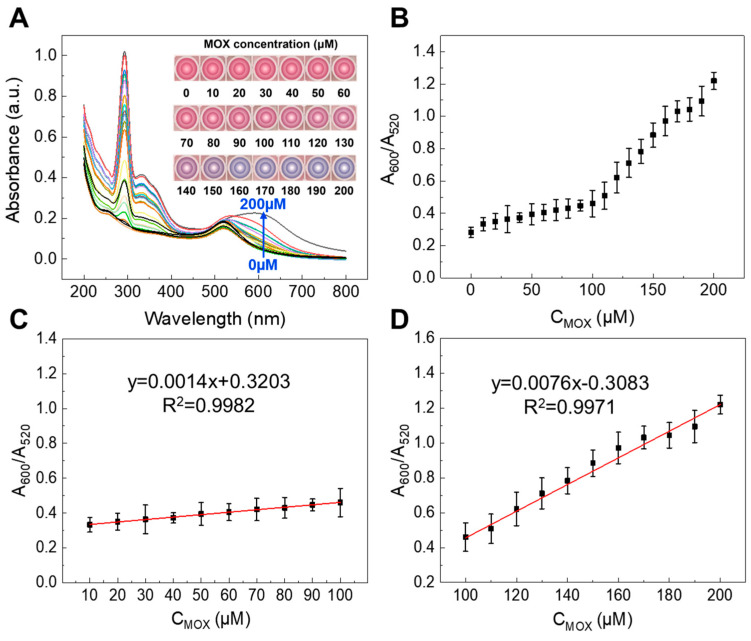
The detection of MOX using the ATG-AuNPs. (**A**) The change in the UV–Vis absorption spectra was recorded for the ATG-modified AuNPs upon increasing the MOX concentration; inset: photos showing the corresponding sample color. (**B**) The relationship between A_600nm_/A_520nm_ and the MOX concentration. The linear calibration curve with different concentrations: (**C**) MOX (0~100 µM) and (**D**) MOX (100~200 µM).

To investigate the sensitivity of the ATG-AuNPs assays for the quantitative detection of MOX/CIP and Cr(III), ATG-AuNPs were mixed with their solutions at different concentrations under optimized conditions. The photograph and UV–Vis absorbance spectra were collected. As the concentration of MOX increased from 0 µM to 200 µM, the color of the ATG-AuNPs gradually changed from wine-red to blue-gray (Figure 5A). The UV–Vis spectra of ATG-AuNPs showed that the characteristic absorbance peaks at 520 nm gradually declined along with increasing MOX concentration. In comparison, the red-shifted absorption band at 600 nm increased gradually. In addition, Figure 5B–D show the linear relationship (y = 0.0014x + 0.3203, y = 0.0076x − 0.3083) between the concentration of MOX ranging from 0 to 200 µM and the logarithm of the A_600/_A_520_ ratio, with correlation coefficients of 0.9982 and 0.9971. The detection limit is 1.57 µM by UV–Vis (S/σ = 3), where S is the slope of the standard curve, and σ is the calibration deviation of the blank.

**Figure 6:**
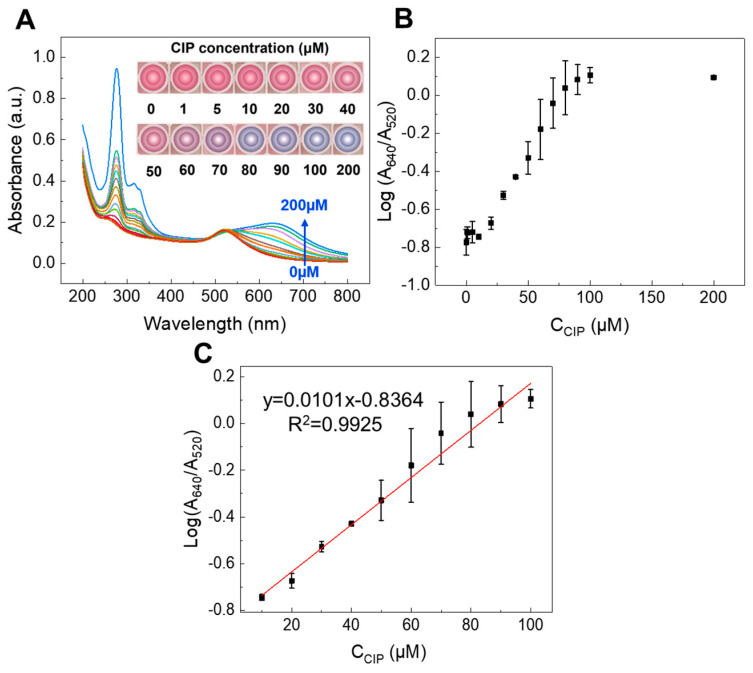
The detection of CIP using the ATG-AuNPs. (**A**) The change in the UV–Vis absorption spectra was recorded for the ATG-modified AuNPs upon increasing the CIP concentration; inset: photos showing the corresponding sample color. (**B**) The relationship between Log(A_640nm_/A_520nm_) and the CIP concentration. (**C**) The linear calibration curve with different concentrations CIP (0~100 µM).

**Figure 7:**
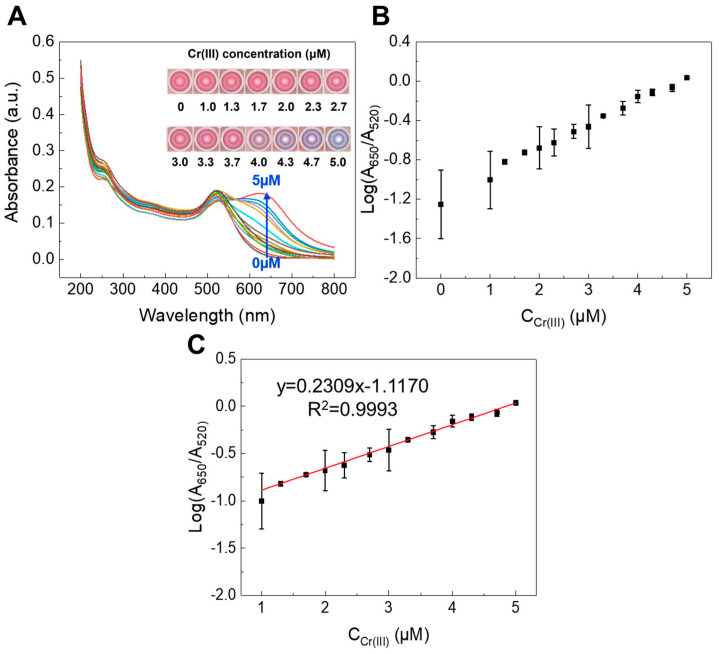
The detection of Cr(III) using the ATG-AuNPs. (**A**) The change in the UV–Vis absorption spectra was recorded for the ATG-modified AuNPs upon increasing the CIP concentration; inset: photos showing the corresponding sample color. (**B**) The relationship between Log(A_650nm_/A_520nm_) and the Cr(III) concentration. (**C**) The linear calibration curve with different concentrations Cr(III) (0~5.0 µM).

Furthermore, with a gradually increasing CIP concentration (0~100 µM) in the ATG-AuNPs solution, the characteristic absorption peak at 520 nm was red-shifted to 640 nm. Meanwhile, a gradual decrease was found in the absorption spectra at 520 nm. In addition, the intensity of the absorption peak of the ATG-AuNPs at 650 nm was notably improved with the gradually increasing Cr(III) concentration (0~10 µM). The corresponding absorbance calibration curve was plotted versus CIP or Cr(III) concentration (Figure 6 and Figure 7). Satisfactory linear relationships were obtained for CIP and Cr(III), with R^2^ = 0.9925 and 0.9993 correlation coefficients, respectively. The detection limit (LOD) of the ATG-AuNPs for CIP and Cr(III) is computed to be 1.30 µM and 57.1 nM, respectively.

Table S1 compares the current methods with previous methods for quantitatively detecting MOX/CIP and Cr(III). It shows that the LOD and dynamic range of the proposed ATG-AuNPs nanosensor are enhanced compared with other reported methods. Thus, based on the above results, the developed method has a comparative or even better performance and can be considered a novel standalone system that can meet the demand for the simple, fast, and sensitive simultaneous detection of MOX/CIP and Cr(III).

#### 3.2.6. Investigation of the Performance of ATG-AuNPs Sensor in Actual Samples

The colorimetric detection capability of ATG-AuNPs in the actual environment was investigated by spiked recovery experiments on complex samples. Two standard practical samples were chosen: tap water and lake water (Table 1). The standard additive approach has been applied to avoid the effects of the matrix. In this way, several analyte concentrations within the calibration curve range were added to the probe with actual samples under optimal conditions. The analysis method was carried out in the same manner as described in the Section 2, and then the results were reported. According to the obtained results, the recoveries obtained were in the range of 96.42~98.77%, 96.85~99.71%, and 97.00~99.54% for MOX, CIP, and Cr(III), respectively, which demonstrated that the ATG-AuNPs probe as an inexpensive and straightforward colorimetric sensor has an outstanding efficiency and performance for the simultaneous detection of MOX/CIP and Cr(III) in actual samples.

Finally, to evaluate the accuracy and precision of the proposed method, repeatability tests were conducted. Intra-day (n = 10) repeatability tests demonstrated the RSD values for MOX, CIP, and Cr(III) were lower than 1.4%, 1.4%, and 4.1%, respectively (Figures S7, S8, and S9). These values indicate that the developed method exhibits excellent accuracy and precision.

## 4. Conclusions

In conclusion, ATG-AuNPs as a colorimetric sensor were prepared using a simple, low-cost, and extensible strategy through the chemical synthesis of AuNPs and subsequently by surface modification with ATG. Transmission electron microscopy (TEM) verified the successful preparation and modification of AuNPs and ATG-AuNPs. Interestingly, after modification by ATG, low levels of three analytes were investigated by the ATG-AuNPs probe, and the sensor’s aggregation and color change were found. Investigating various parameters indicated that the maximum response of the ATG-AuNPs sensor for detecting MOX/CIP and Cr(III) was obtained in 80 µM and 1 min. The results confirmed that the ATG-AuNPs sensor’s sensitivity was high, and its detection limit was observed at 1.57 µM, 1.03 µM, and 57.1 nM for MOX, CIP, and Cr(III), respectively. The recovery by utilizing this sensor in an actual sample was observed to be in the range of 96.42~98.77%, 96.85~99.71%, and 97.00~99.54% for MOX/CIP and Cr(III), which demonstrated that the ATG-AuNPs probe as a simple, rapid, and inexpensive colorimetric sensor has an outstanding performance and efficiency for simultaneously determining MOX/CIP and Cr(III) in actual samples.

## Figures and Tables

**Table 1:** Practical application of developed sensor with tap and lake water.

	Samples	Added (μM)	Tap Water			Lake Water		
			Measured (μM)	Recovery (%)	RSD (%)	Measured (μM)	Recovery (%)	RSD (%)
	1	20	19.29	97.87	3.40	19.35	96.77	3.40
MOX	2	30	28.92	96.42	3.53	29.59	98.77	3.34
	3	40	38.95	97.38	3.07	38.93	97.33	2.20
	1	20	19.61	98.05	0.46	19.37	96.85	1.96
CIP	2	30	29.84	99.46	2.50	29.89	99.62	3.30
	3	40	39.85	99.63	1.30	39.88	99.71	2.70
	1	1	1.00	99.54	2.37	0.99	99.08	0.93
Cr(III)	2	2	1.95	97.70	2.70	1.94	97.00	0.40
	3	3	2.98	99.50	1.60	2.93	97.62	1.10

## Data Availability

The datasets used and/or analyzed during the current study are available from the corresponding author upon reasonable request.

## Supplementary Materials

The following supporting information can be downloaded at https://www.mdpi.com/article/10.3390/s25103228/s1, Electronic Supplementary Information (ESI) available: Experimental figures.
