# Supplementary material for: An Ultra-Sensitive Colorimetric Sensing Platform for Simultaneous Detection of Moxifloxacin/Ciprofloxacin and Cr(III) Ions Based on Ammonium Thioglycolate Functionalized Gold Nanoparticles
Source: Sensors (Basel). 2025 May 21;25(10):3228. doi: 10.3390/s25103228 (PMC12116012; doi:10.3390/s25103228)
Supplement: Supplementary file 1 [file sensors-25-03228-s001.zip › sensors-3619701-supplementary.pdf]

Supporting Information

of

An Ultra-Sensitive Colorimetric Sensing Platform for Simultaneous Detection of Moxifloxacin/Ciprofloxacin and Cr(III) Ions Based on Ammonium Thioglycolate-Functionalized Gold Nanoparticles

Lihua Zhang [1], Jiang Li [1], Juan Wang [1], Xu Yan [1], Jinping Song [2], Feng Feng [1,*]

[1] Shanxi Provincial Key Laboratory of Chemical Biosensing, School of Chemistry and Chemical Engineering, Shanxi Datong University, Datong 037009, China

[2] Department of Chemistry, Xinzhou Normal University, Xinzhou, Shanxi, 034000, China.

* Corresponding author: Feng Feng, E-mail: feng-feng64@263.net;

Table of Contents

Figures.. S-2

Figure S1. UV-vis absorbance spectra and the absorbance at 520 nm recorded for the stability of the ATG-AuNPs solution stored at 4 °C at different times.. S-3

Figure S2. The UV-Vis absorption of the AuNPs in the presence of different concentrations of ATG (0~1000 μM) S-4

Figure S3. The UV-Vis absorption spectra of ATG-modified AuNPs solutions with MOX reaction time from 0 to 60 min... S-5

Figure S4. The UV-Vis absorption spectra of ATG-modified AuNPs solutions with CIP reaction time from 0 to 60 min... S-6

Figure S5. The UV-Vis absorption spectra of ATG-modified AuNPs solutions with Cr(III) reaction time from 0 to 60 min... S-7

Figure S6. Anti-interference test for the detection system in the presence of MOX and Cr(III). S-8

Figure S7. Anti-interference test for the detection system in the presence of MOX and CIP. S-9

Figure S8. Repeatability test of the detection system for MOX analysis.. .... S-10

Figure S9. Repeatability test of the detection system for CIP analysis.. .... S-11

Figure S10. Repeatability test of the detection system for Cr(III) analysis.................. S-12

Table S1. Comparison between previously reported sensors for detecting MOX/CIP and Cr(III). S-13

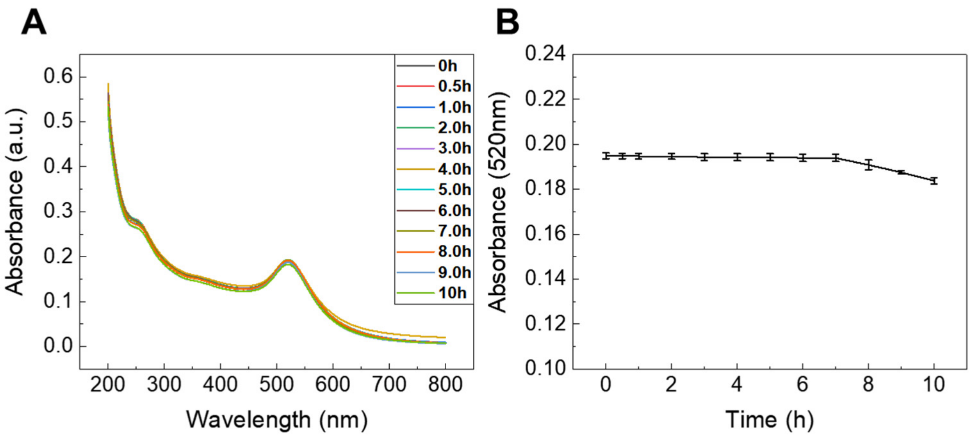

Figure S1. (A) UV-vis absorbance spectra and (B) the absorbance at 520 nm recorded for the stability of the ATG-AuNPs solution stored at 4 °C at different times.

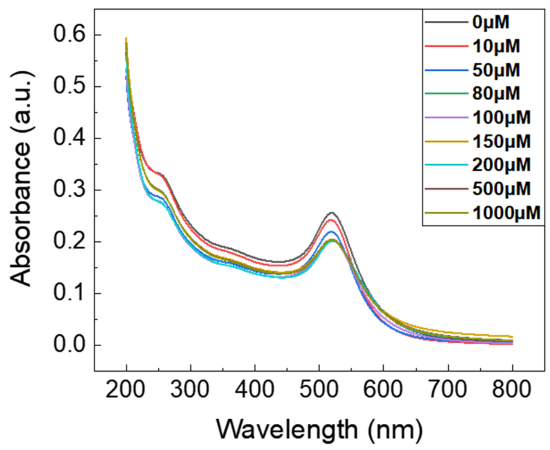

Figure S2. The UV-Vis absorption of the AuNPs in the presence of different concentrations of ATG (0~1000 μM).

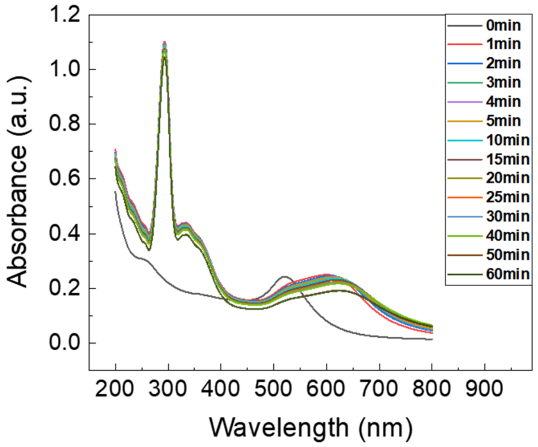

Figure S3. The UV-Vis absorption spectra of ATG-modified AuNPs solutions with MOX reaction time from 0 to 60 min. The MOX concentration was 200 μM.

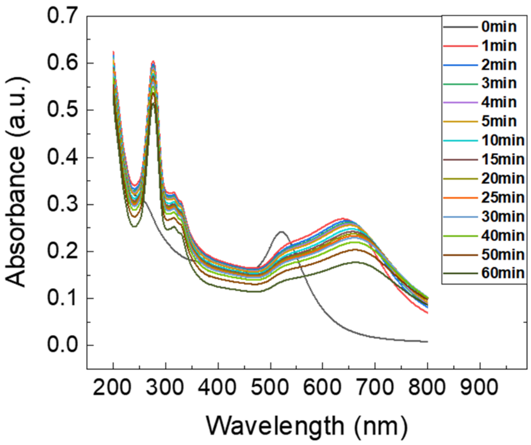

Figure S4. The UV-Vis spectra of ATG-modified AuNPs solutions with CIP reaction time from 0 to 60 min. The CIP concentration was 100 μM.

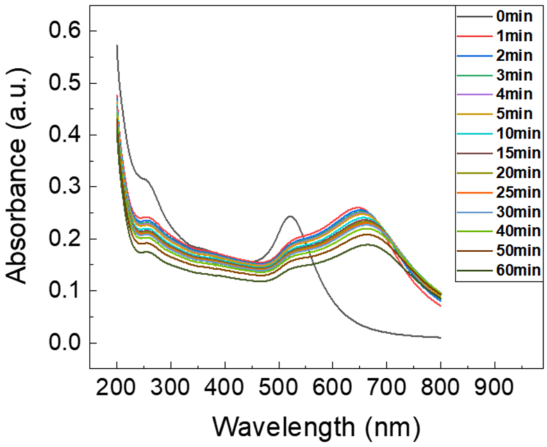

Figure S5. The UV-Vis spectra of ATG-modified AuNPs solutions with Cr(III) reaction time from 0 to 60 min. The Cr(III) concentration was 10 μM.

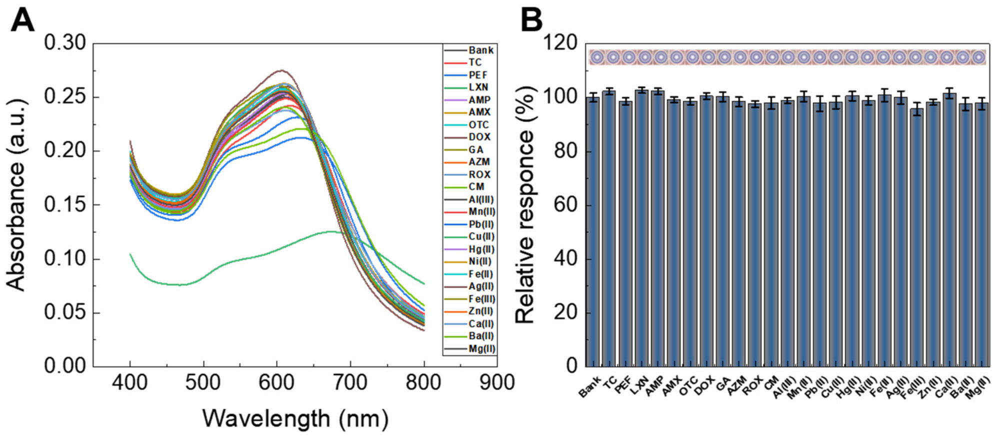

Figure S6. Anti-interference test for the detection system. (A) UV-vis absorption spectra and (B) the relative response for the ATG-AuNPs system in the presence of MOX/Cr(III) and a mixture of MOX/Cr(III) and other potential targets; inset: photos showing the corresponding sample color. (MOX, CIP, TC, PEF, LXN, AMP, AMX, OTC, DOX, GA, AZM, ROX, CM are 200 μM, respectively) and (Cr(III), Al(III), Mn(II), Pb(II), Cu(II), Hg(II), Ni(II), Fe(II), Ag(I), Fe(III), Zn(II), Ca(II), Ba(II) and Mg(II) are 10 μM, respectively).

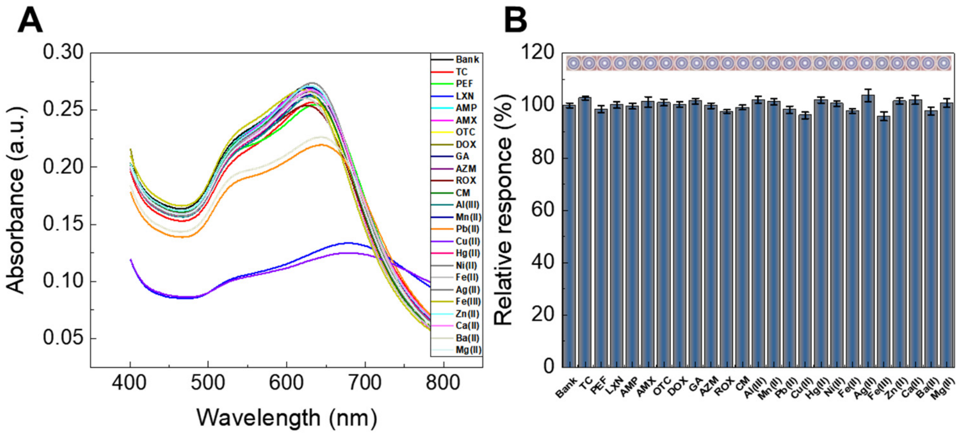

Figure S7. Anti-interference test for the detection system. (A) UV-vis absorption spectra and (B) the relative response for the ATG-AuNPs system in the presence of MOX/CIP and a mixture of MOX/CIP and other potential targets; inset: photos showing the corresponding sample color. (MOX, CIP, TC, PEF, LXN, AMP, AMX, OTC, DOX, GA, AZM, ROX, CM are 200 μM, respectively) and (Cr(III), Al(III), Mn(II), Pb(II), Cu(II), Hg(II), Ni(II), Fe(II), Ag(I), Fe(III), Zn(II), Ca(II), Ba(II) and Mg(II) are 10 μM, respectively).

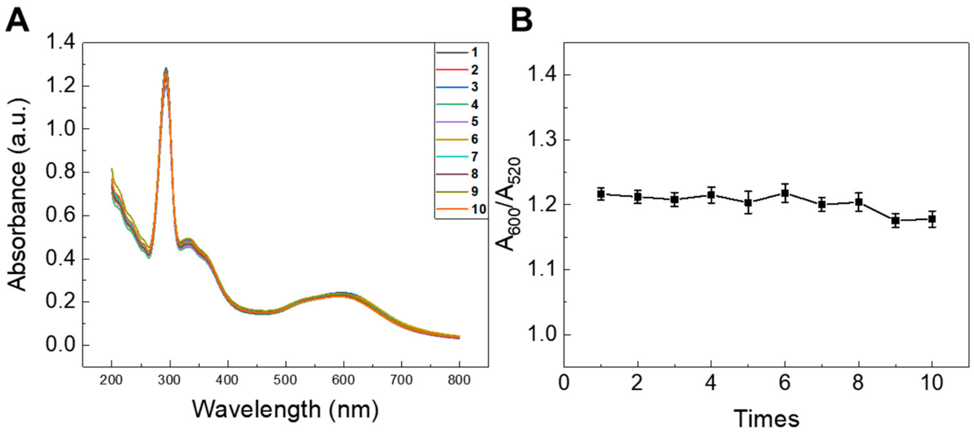

Figure S8. Repeatability test for the detection system. (A) The UV-Vis spectra and (B) of ATG-modified AuNPs solutions following reaction with MOX, with the experiment repeated 10 times. The MOX concentration was 200 μM.

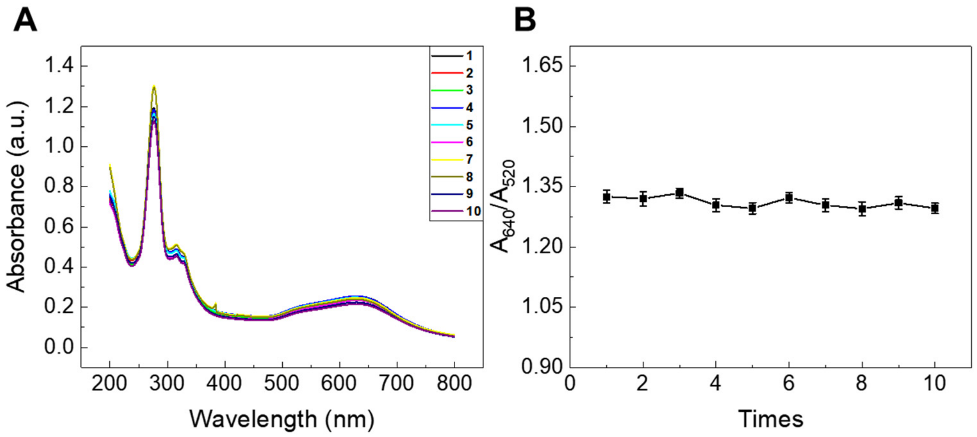

Figure S9. Repeatability test for the detection system. (A) The UV-Vis spectra and (B) of ATG-modified AuNPs solutions following reaction with CIP, with the experiment repeated 10 times. The CIP concentration was 200 μM.

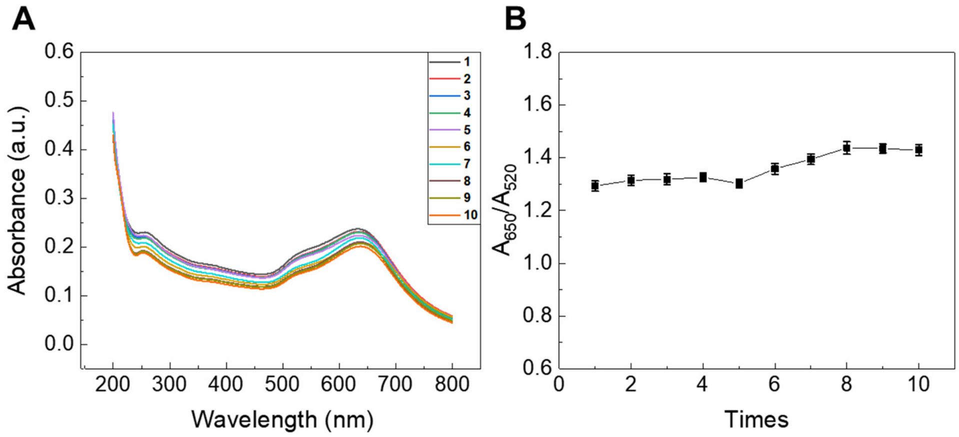

Figure S10. Repeatability test for the detection system. (A) The UV-Vis spectra and (B) of ATG-modified AuNPs solutions following reaction with Cr(III), with the experiment repeated 10 times. The Cr(III) concentration was 10 μM.

Table S1 Comparison between previously reported sensors for detecting MOX/CIP and Cr(III).

	Techniques	Media/ Nanomaterials	Linear range (μM)	Lower detection limit (μM)	Real Samples	Ref.
MOX	HPLC	_	0.311-39.85	0.311	Human Plasma	12
	LC-MS	_	0.0125-0.249	0.0125	Rat Plasma	17
	Electrochemical	MIP/Au/PEDOT/GCE	0.004-20.0	0.0011	Milk, Honey and Moxifloxacin preparations	21
	AAS	0.01 M hydrochloric acid + ammonium reineckate	99.64-1096.1	3.7367	MOX in its pure form, Avalox® Tablets (400mg/tablet) and Urine	24
	Fluorescence	PVA synthesized CQDs	0.025-15.0	0.0063	Milk and Egg	26
	Colorimetric	MPBA-AgNPs	0.05-1.0	0.27	Human Serum	46
	Colorimetric	ATG-AuNPs	10.0-200.0	1.57	Tap water and Lake water	This work
CIP	HPLC	_	1.177-150.898	0.332	Pharmaceutical Formulations	13
	LC-MS	_	0.0302-2.263	0.0151	Aquatic Matrices	16
	Electrochemical	HKUST-1/$CoFe_2O_4$/g-C_3N_4	0.05-180.0	0.0026	Serum and Urine	22
	Fluorescence	CD-LnPOM	0.0-100.0	0.0003	River water	27
	Colorimetric	PAM@AuNPs-TMB-H_2O_2	1.0-12.0	0.5	Rat Serums	36
	Colorimetric	PUE-AuNPs	1.0-1000.0	51	Tap water and Milk	37
	Colorimetric	ATG-AuNPs	10.0-100.0	1.30	Tap water and Lake water	This work
Cr(III)	ICP-MS	2-Nitroso-1-naphthol impregnated MCI GEL CHP20P	_	0.0008	CRMs and Natural water	20
	Electrochemical	DNAzymes immobilized on Pt nanoparticles for non-faradaic EIS	0.005-0.2	0.001	Tap water	23
	Fluorescence	CS-TP-50	1.0-50.0	0.0446	Soil water, Rainwater and Lake water	28
	Colorimetric	F-AuNPs	1.0-30.0	0.105	Tap water	38
	Colorimetric	4-MBA-AuNPs	20.0-25.0	5	Simulated samples	39
	Colorimetric	ATG-AuNPs	1.0-5.0	0.0579	Tap water and Lake water	This work
